# Total Flavonoids of *Rhizoma Drynariae* Ameliorate Bone Growth in Experimentally Induced Tibial Dyschondroplasia in Chickens *via* Regulation of OPG/RANKL Axis

**DOI:** 10.3389/fphar.2022.881057

**Published:** 2022-05-26

**Authors:** Tingting Xu, Jingjing Zheng, WeiXing Jin, Lu Li, Luxi Lin, Aftab Shaukat, Chaodong Zhang, Qinqin Cao, Muhammad Ashraf, Shucheng Huang

**Affiliations:** ^1^ College of Veterinary Medicine, Henan Agricultural University, Zhengzhou, China; ^2^ Sanquan College of Xinxiang Medical University, Xinxiang, China; ^3^ National Center for International Research on Animal Genetics, Breeding and Reproduction (NCIRAGBR), Huazhong Agricultural University, Wuhan, China; ^4^ Livestock and Dairy Development Department, Pishin, Pakistan

**Keywords:** bone development, Chinese herbal medicine, leg disease, tibial dyschondroplasia, total flavonoids of *Rhizoma Drynariae*

## Abstract

**Background:** *Rhizoma Drynariae*, traditional Chinese herb, is widely used to treat and prevent bone disorders. However, experimental evidence on the use of *Rhizoma Drynariae* extract, total flavonoids of *Rhizoma Drynariae* (TFRD) to treat tibial dyschondroplasia (TD) in chickens and its underlying mechanisms have not been investigated.

**Purpose:** To evaluate the therapeutic effect of TFRD on leg disease caused by TD and elucidate its mechanisms in modulating the bone status.

**Methods:** Thiram-induced chicken TD model has been established. The tibia status was evaluated by analyzing tibia-related parameters including tibial weight, tibial length and its growth plate width and by performing histopathological examination. The expression of tibial bone development-related genes and proteins was confirmed by western blotting and qRT-PCR.

**Results:** The results showed that administration of TFRD mitigated lameness, increased body weight, recuperated growth plate width in broilers affected by TD and the increase of tibia weight and tibia length is significantly positively correlated with body weight. Compared with the TD group broilers, 500 mg/kg TFRD evidently reduced the damage width of the growth plate and improved its blood vessel distribution by elevating the gene expression levels of BMP-2 and Runx2 and OPG/RANKL ratio. Furthermore, correlation analysis found that the damage width of the growth plate was negatively correlated with the expression levels of BMP-2 and OPG.

**Conclusion:** The present study revealed that TFRD could promote the bone growth *via* upregulating OPG/RANKL ratio, suggesting that TFRD might be a potential novel drug in the treatment of TD in chickens.

## Introduction

Tibial dyschondroplasia (TD) is a commonly occurring leg problem in fast growing birds caused by vascular injury or ischemia resulting in abnormal chondrocyte differentiation in the proximal tibial growth plate, in which leads to the claudication and brittle fracture (Huang et al., 2017, 2018; Zhang et al., 2018). The development of growth plate (GP) includes integrated processes of chondrocyte differentiation, proliferation, mineralization and osteogenesis (Lui et al., 2014). Among them, the vascularization of the GP is essential for the proliferation and differentiation of chondrocytes to promote bone mineralization (Huang et al., 2018). However, it is noted that non-vascularized, non-mineralized white opaque masses are generally observed during GP development in TD broilers (Zhang et al., 2018). TD broilers are usually characterized by difficulty in standing, inflexible gait, limited intake of food and water, retarded growth and slow weight gain, which cause a decrease in production performance (Huang et al., 2018).

The broiler chickens with TD can also reduce the meat yield and quality, thereby causing huge economic losses to the poultry industry (Huang et al., 2019a; Huang et al., 2021a). Although there is a lot of evidence that angiogenesis dysfunction (Huang et al., 2018; Huang et al., 2019b), genetic selection (Kapell et al., 2017), manganese deficiency (Dong et al., 2021), calcium and phosphorus metabolism disorders (Ledwaba and Roberson 2003), and dietary dithiocarbamates (Rath et al., 2007; Huang et al., 2019a) plays a role in the establishment of TD lesions. However, the cause and pathogenesis of TD lesion formation in chickens and how to prevent and minimize its harmful effects on the poultry industry remains the challenge for future research.

Previous studies demonstrated that the occurrence of TD in broilers is related to the abnormal development of tibial bone (Huang et al., 2017). Bone morphogenetic protein-2 (BMP-2) is an important factor regulating bone formation that promotes the differentiation of bone marrow mesenchymal stem cells into osteoblasts (Song et al., 2014). Moreover, many studies have revealed that most members of BMP can promote the growth and development of osteoblasts (Feng and Derynck, 2005; Bruderer et al., 2014). BMP-2 binds to cell surface receptors during bone formation and activates downstream signaling molecules called Smads, Smads (including Smad1, Smad5 and Smad8), which are activated and combined with Smad4 to form Smads complex. It is then transferred into the nucleus as transcriptional enhancers by activating runt-related transcription factor-2 (Runx2) and promoting osteoblast differentiation and maturation (Nuschke et al., 2014; Pierrefite-Carle et al., 2015). In recent years, osteoprotegerin (OPG)/receptor activator of nuclear factor kappa-B ligand (RANKL) axis has been extensively studied that the high expression of OPG and low expression of RANKL can effectively promote the expression of osteoblasts and accelerate the recovery of bone in through (Ibáñez, et al., 2019; Amin, et al., 2020; Ma, et al., 2020), which may be through the combination of OPG and the key factor RANKL in the process of osteoclast differentiation to inhibit the combination of RANKL and RANK, thereby blocking the differentiation of osteoclast precursors into osteoclasts (Udagawa et al., 2021). However, the underlying mechanism of OPG/RANKL axis to regulate tibial development in TD broilers remains largely unknown.

Recently, the incidence of broiler leg disease can be reduced by immunization, reasonably increasing stocking density, nutrition management and light time (Garner et al., 2012; Kierończyk et al., 2017), which is not expected to increase production costs and labor. In addition, it is essential to select low-residue drugs for prevention and treatment due to the short growth cycle and long drug metabolism time of broilers. Traditional Chinese medicine (TCM) has a long history, less harmful ingredients, fewer side effects, lesser residues, and is relatively safe (Zhang et al., 2016, 2018; Sun et al., 2020). Therefore, TCM may become the first choice for the prevention and treatment of various bone disorders including TD (Yao et al., 2018). Total flavonoids from *Rhizoma Drynariae* (TFRD), a Chinese herbal medicine product was extracted from the root of *Drynaria roosii Nakaike* (Chen et al., 2021). TFRD can replenish the kidney, strengthen the bones, promote the healing of fracture, and relieve pain. It has been proved to be useful in the treatment and prevention of bone disorders by changing the function of articular cartilage cells, increasing the proliferation and differentiation of osteoblasts, and promoting bone strength (Song et al., 2016; Zhang et al., 2017; Yao et al., 2018). Therefore, the present study aimed to investigate whether TFRD has the therapeutic and protective effects on tibial development of TD broiler chickens via regulating the OPG/RANKL axis.

## Materials and Methods

### Animal Ethics and Experimental Materials

All experimental procedures were conducted strictly following the Guidelines of the Care and Use of Laboratory Animals in China. This study was approved by the ethics committee of Henan Agricultural University, Zhengzhou, China (Permit No: 17-0126).

All Arbor Acres (AA) chickens (regardless of gender; 1-day-old; 48.25 ± 1.15 g) were obtained from a commercial hatchery (Xingda Poultry Industry Co., Ltd., Kaifeng, China). Thiram (AR, purity ≥98%, #C10036460) was acquired from Macklin Biochemical Co., Ltd. (Shanghai, China). TFRD (#K198752, purity ≥95%) was detected by HPLC and purchased from Xi’an Kailai Biological Engineering Co., Ltd. (Xi’an, China).

### Experimental Design

All broiler chickens were divided randomly into six separate groups (*n* = 40/group, 10 chicks per replicate and four replicates per group): the control group (Con), thiram-induced TD group (TD), TFRD prevention group (LTFRD), dose gradient TFRD-treated group (TD + LTFRD, TD + MTFRD and TD + HTFRD). The management protocols used for the broilers in each group was as follows: Broilers in the Con group were fed a normal diet, the LTFRD group were fed a normal diet with 125 mg/kg TFRD, and the experimental groups (TD, TD + LTFRD, TD + MTFRD, and TD + HTFRD) were fed the same diet as the Con group supplemented with 100 mg/kg thiram from day 4 to day 7. For TFRD-treated groups including TD + LTFRD, TD + MTFRD, and TD + HTFRD group, all broiler chickens were administered with 125 mg/kg, 250 mg/kg, 500 mg/kg of the TFRD in fed during the entire experimental period. The dosage of TFRD were based on previous reports (Yao et al., 2018; Lv et al., 2021). All broilers were housed in a controlled environment maintained at the recommended standard room temperature (23–35°C), relative humidity (60–70%), 12:12 h dark or light cycle, ventilation, and hygienic conditions as required for broilers.

### Sample Collection and Analysis of Tibia-Related Parameters

All groups were reared for 21 days, and daily body weight gain in every group was calculated. Ten chicks per group were randomly selected and euthanized on days 7, 14, and 21. Then, the removal of the shank muscles and the exposure of the tibia growth plate region was performed. In addition, the bone histomorphometric parameters, such as the weight, length, and the tibia growth plate (TGP) width were determined by an electronic balance with a sensitivity of 0.001 g (#FA1204N, Jinghai Instrument Co., Ltd., Shanghai, China) and digital calipers (#SATA91511, TATA Company, Shanghai, China). Then the tibia bone sections were fixed in paraformaldehyde (4%) for hematoxylin and eosin (H&E) staining. The TGPs were frozen in liquid nitrogen and stored at −80°C for further analysis, such as quantitative real-time polymerase chain reaction (RT-qPCR) and western blotting.

### Flavone Content Measurement

The flavone was analyzed by HPLC at BioNovoGene (Suzhou, China) (http://www.bionovogene.com/). Analysis of the samples was performed using liquid chromatography-mass spectrometry (LC-MS), carried out on a Waters ACQUITY UPLC equipped with an AB 4000 Triple Quadrupole Mass Spectrometer (AB 4000). The chromatographic conditions were as follows: ACQU ITY UPLC® BEH C18 column (2.1 × 100 mm, 1.7 μm, Waters, United States), injection volume 5 μl, column temperature 40°C, mobile phase A1-0.1% formic acid water, mobile phase B- Methanol, flow rate 0.25 ml/min. The mass spectrometry conditions were as follows: Electrospray ionization (ESI) source, negative ion ionization mode. The ion source temperature was 500°C, the ion source voltage was −4,500 V, the collision gas was 6 psi, the curtain gas was 30 psi, and the atomizing gas and auxiliary gas were both 50 psi. Scans were performed using multiple reaction monitoring (MRM). The samples were quantified according to the standard curve (Supplementary Table S1).

### Hematoxylin and Eosin Staining

The tibia samples from each group were fixed in 4% paraformaldehyde. The paraffin sections were prepared by conventional methods, and then dewaxed in xylene, dehydrated in graded ethanol solutions and embedded in paraffin. Tibia section (5 μm) were stained with hematoxylin and eosin (H&E), and examined by optical microscopy using a Motic BA600-4 microscope (Motic^Ⓡ^, Xiamen, China) to assess tibial histopathology.

### RNA Isolation and qRT-PCR

Total RNA of the tibia sample was extracted using Trizol reagent (China Biological Engineering Co., Ltd., China). The integrity of total RNA was checked on denaturing formaldehyde agarose gel and the concentration of RNA was quantified by Nanodrop 2000 (Thermo scientific) spectrophotometer. Primers specific for GAPDH (Glyceraldehyde-3 phosphate dehydrogenase), OPG, RNAKL, BMP-2 and Runx2 were designed by Premier software (#Version 5.0., Canada) and synthesized by Tsingke Biotechnology Co., Ltd. (Zhengzhou, China). All the primer sequences in this study are shown in Table 1. GAPDH was used as a loading control. The qRT-PCR was performed according to the SYBR Green I PCR Master Mix (Vazyme Biotech Co., Ltd., China). The PCR amplification thermal cycling consisted of predeformation at 95°C for 30 s, followed by 40 cycles consisting of denaturation at 95°C for 10 s, extension at 72°C for 30 s, and annealing at 60°C for 30 s. Calculated according to the expression of mRNA using the 2^−ΔΔCT^ method (Lin et al., 2022). All the assays were performed three times to ensure accuracy.

**TABLE 1:** Primers used for the quantitative polymerase chain reaction.

Genes	Gene bank ID	Primer sequence (5′-3′)	Products length
*Runx2*	AF_445419	F: TAA​AGG​TGA​CGG​TGG​ATG​G	190
		R: TGT​GGA​TTA​AAA​GGA​CTT​GGT​G	
*OPG*	NM_001033641.1	F: ACAGGGACCGCAACAAGT	122
		R: CAGCCGAGTGCTCTGACT	
*RANKL*	AB175678.1	F: CGG​AGG​ATA​TGA​TGT​TCA​C	79
		R: TAG​GAG​GGC​ACA​GAA​TAA​C	
*BMP-2*	XM_015283435.1	F: TCA​GCT​CAG​GCC​GTT​GTT​AG	185
		R: ACC​CCA​CGT​CAT​TGA​AGT​CC	
*GAPDH*	NM_204305.1	F: GCCCAGAACATCATCCCA	137
		R: CGGCAGGTCAGGTCAACA	

Note: F, forward primer; R, reverse primer.

### Western Blot Analysis

Tibial growth plates from all the groups were initially homogenized in PBS (freezing-cold) solution and kept at 4°C for 2 h. After that, the homogenate was centrifuged at 14,000 rpm for 10 min to collect the total protein. Coomassie Brilliant Blue G-250 method was used to calculate the content of total protein (Zhang et al., 2018). Moreover, protein from the samples was separated with the help of SDS polyacrylamide gel and then moved to polyvinylidene fluoride (PVDF) membranes, which were incubated for 2 h in 5% skimmed milk. Next, membranes were incubated overnight at 4°C with primary antibodies against OPG (#0431R, 1:1,000, Bioss, Beijing, China), RANKL (#0747R, 1:1,000, Bioss, Beijing, China), and β-actin (#WL01372, 1:1,000, Wanleibio, Shenyang, China). Subsequently, membranes were washed extensively using Tris-buffered saline, containing 0.1% Tween 20 (TBST) for 30 min and then incubated for 2 h at room temperature with secondary antibody (HRP goat anti-rabbit IgG, #WLA023a, 1:5,000 dilution; Wanleibio, Shenyang, China). Later, with the help of TBST, membranes were washed 30 min again rigorously, and images were taken with an imaging system (AI600, CE, United States). At the end, gray level of the exposed strip was analyzed.

### Statistical Analysis

All of the statistical analyses were performed with SPSS. Software (#version 26.0, SPSS Inc., Chicago, IL, United States) by using one-way ANOVA followed by the LSD test as the post-hoc test. The correlation analysis between body weight and tibial parameters, tibia growth parameters and bone formation, and bone resorption-related indices were performed by Prism statistical analysis software (#Version 8.00, GraphPad Software, Inc.). The PCA analysis of bone resorption and bone formation-related genes through genetic cloud tools (a free online data analysis platform, https://www.omicstudio.cn). All experiments were performed at least in triplicates with data expressed as mean ± standard deviation (SD), and it was considered that *p* < 0.05 represents statistically significant.

## Results

### Effects of Total Flavonoids of *Rhizoma Drynariae* on Tibia Growth in Broilers With Tibial Dyschondroplasia

**FIGURE 1:**
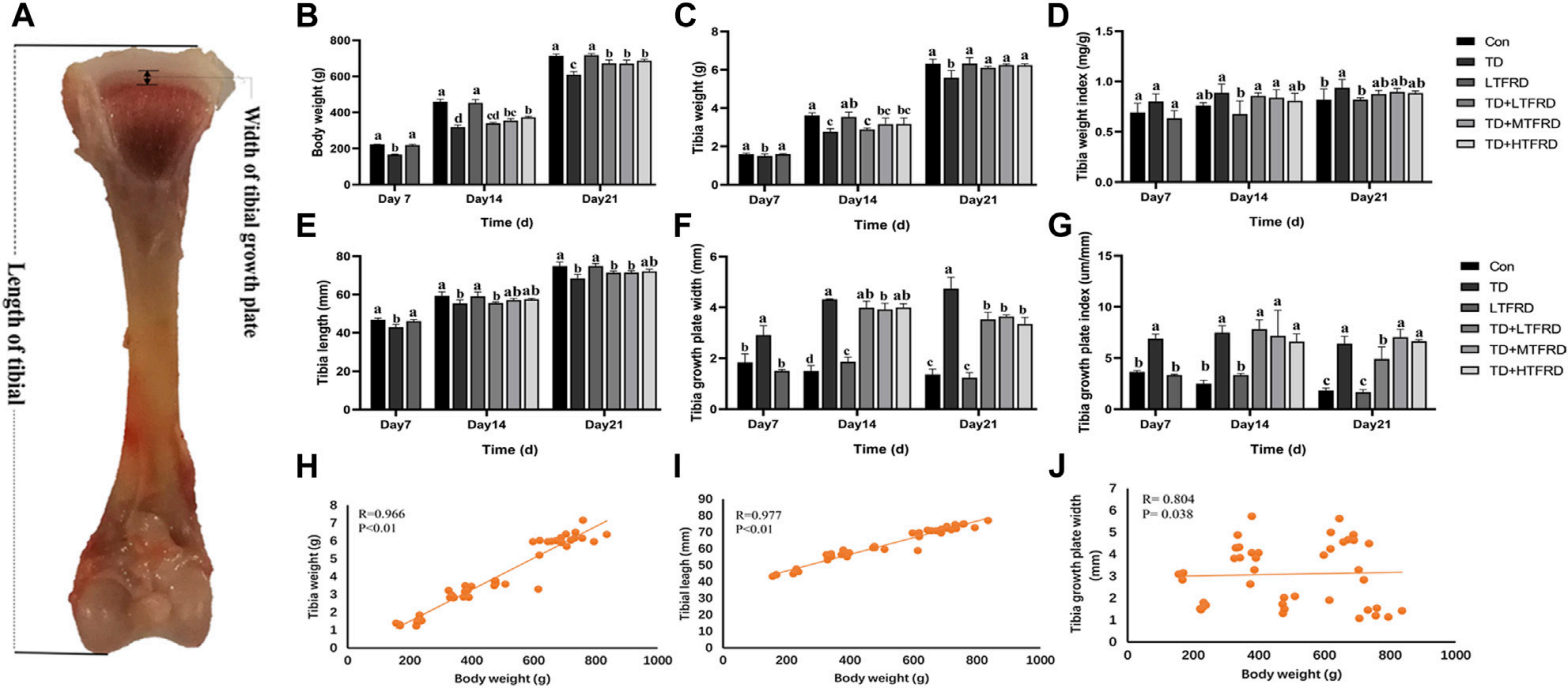
Effects of TFRD on tibial growth in TD broilers. **(A)** The tibial of chickens. **(B)** Body weight of chickens. **(C)** Tibia weight. **(D)** Tibia weight index. **(E)** Tibia length. **(F)** Tibia growth plate width. **(G)** Tibia growth plate index. **(H)** The pearson correlation analysis between tibia weight and body weight. **(I)** The pearson correlation analysis between tibia length and body weight. **(J)** The pearson correlation analysis between tibia growth plate width and body weight. The results are represented as mean ± SD. a, b, c, and d represent significant differences between groups (*p* < 0.05).

The tibia growth indexes of chickens are presented in Figure 1. The body weight and tibia weight of the TD group were significantly lower than those of the Con group (*p* < 0.001 and *p* < 0.001, respectively). In addition, no statistically difference in body weight and tibia weight have been observed between LTFRD and Con groups on day 7 (*p* = 0.172 and *p* = 0.857, respectively), day 14 (*p* = 0.556 and *p* = 0.941, respectively), day 21 (*p* = 0.828 and *p* = 0.668, respectively) (Figures 1B,C). The TFRD treatment group displayed a good therapeutic effect in body weight and tibia weight, significantly different from the TD group, especially in the high-dose TFRD group. The increase of tibia weight index in TD group also reflected the growth retardation of TD broilers, whereas TFRD supplementation could alleviate the increase of tibia weight index caused by TD (Figure 1D).

As shown in Figures 1E–G, the tibia length was significantly decreased and the TGP width was markedly increased in the TD group compared with the Con group on day 7 (*p* = 0.042 and *p* = 0.004, respectively), day 14 (*p* = 0.008 and *p* < 0.001, respectively) and day 21 (*p* < 0.001 and *p* < 0.001, respectively). TFRD treatment can ameliorate TD-induced abnormality of these two indicators and has the best effect on day 21. The higher the TGP index for TD broilers, the more severe the tibial damage. In the present study, the TGP index in the TD group was higher than that in the Con group, indicating that the tibia in TD broilers was seriously damaged. Conversely, TFRD supplementation can effectively reverse the increase in the TGP index. These results showed that TFRD supplementation alleviated tibia length, TGP width, and TGP index in TD broilers.

Then we conducted a correlation analysis between tibia-related parameters and bodyweight of broilers. As shown in Figure 1, the bodyweight of broilers was significantly positively correlated with tibia length (*p* < 0.01, r = 0.997) and tibia weight (*p* < 0.01, r = 0.966). The body weight was weakly correlated with TGP width (*p* = 0.038, r = 0.804) (Figures 1H–J). The above results indicated that TFRD could promote the tibia growth of TD broilers.

### Effects of Total Flavonoids of *Rhizoma Drynariae* on Morphology and Histology of the Tibia in Broilers With Tibial Dyschondroplasia

**FIGURE 2:**
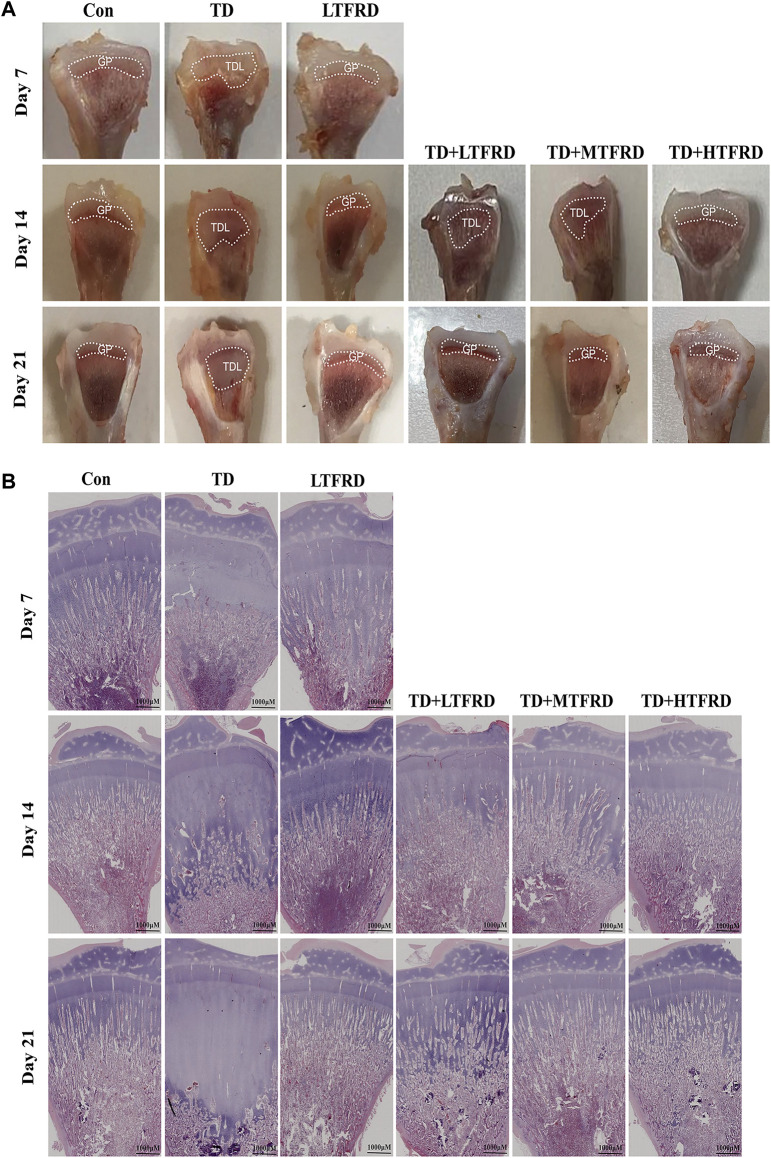
Effects of TFRD on growth plate of tibia bone in TD broilers. **(A)** The morphology result of tibial growth plate. GP, growth plate; TDL, tibial dyschondroplasias lesion. **(B)** The HE staining observation result of tibial growth plate. Scar bar = 1,000 µm.

The results of tibia morphology and histology on the tibia of broilers revealed that the TGP width of LTFRD group was not abnormal, and the arrangement of bone trabecula was normal as compared to the Con group (Figures 2A,B). Additionally, the growth plate of the tibia in the TD group became thicker, and the structure of the trabecular bone was destroyed and broken as compared to the Con group. Especially on day 21, the growth plate width of tibia in the TD group became abnormally thick. After treatment with different doses of TFRD, it was found that the growth plate width of TD + LTFRD group, TD + MTFRD group and TD + HTFRD group is gradually restored to normal when compared with TD group, and the arrangement of bone trabeculae is also becoming orderly, especially on day 21. These results showed that TFRD could restore TD injury of broiler chickens by improving the structure of the TGP.

### Effects of Total Flavonoids of *Rhizoma Drynariae* on Vascular Distribution of the Tibia Growth Plate in Broilers With Tibial Dyschondroplasia

**FIGURE 3:**
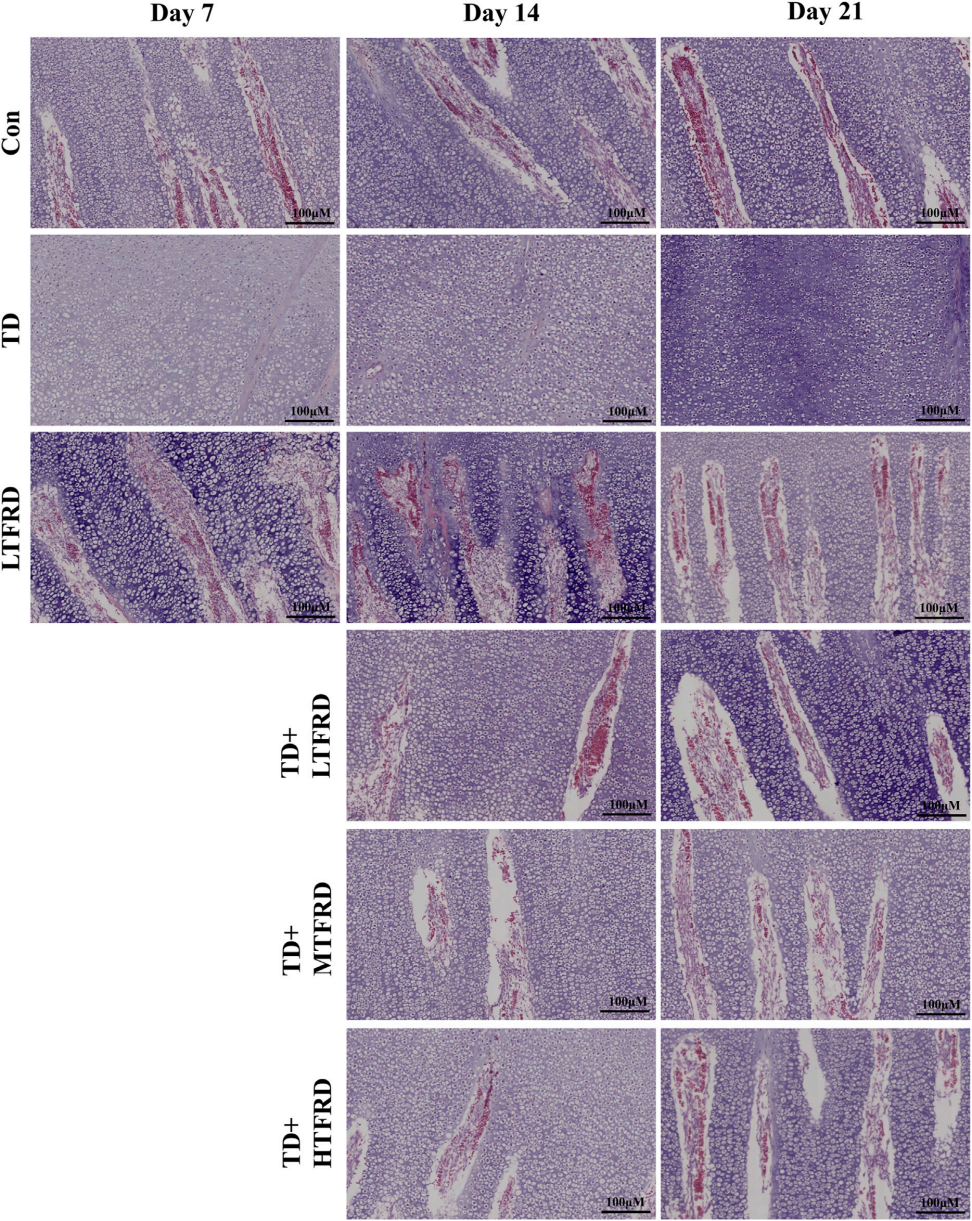
Effects of TFRD on vascular invasion of tibial growth plate in TD broilers. HE staining was used to observe the vascular infiltration of growth plates in each group. The black arrow points to the blood vessel. Scar bar = 100 µm.

Bone formation is dependent upon the richly vascular system. In the present study, the blood vessels in the TGP area of the LTFRD group were more abundant than those in the Con group (Figure 3). It is noted that there is no invasion of blood vessels in the corresponding area of the tibia in the TD group, suggesting that the growth of the tibia in TD broilers may be inhibited (Figure 3). Compared with the TD group, after 2 weeks of intervention with TFRD, the blood vessels in the TGP area of broilers in the TD + LTFRD, TD + MTFRD and TD + HTFRD groups were obviously increased (Figure 3). Collectively, the above findings indicated that TFRD treatment could enhance vascular infiltration in TGP area in TD broilers to a certain extent.

### Effects of Total Flavonoids of *Rhizoma Drynariae* on Gene Expression Levels of *BMP-2/Runx2* and *OPG/RANKL* Pathways of TGP in Broilers With Tibial Dyschondroplasia

**FIGURE 4:**
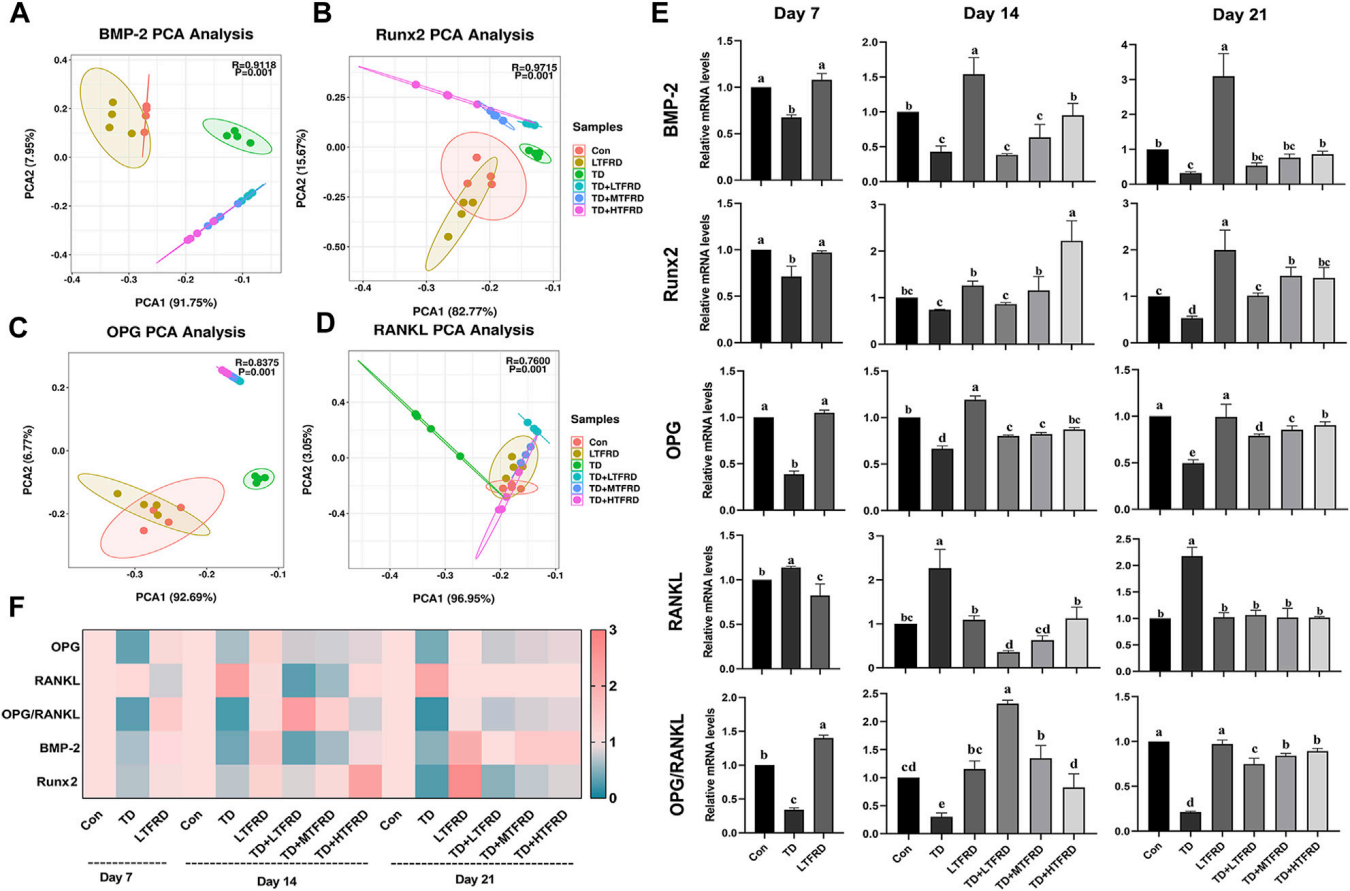
Effects of TFRD on gene expression levels of BMP-2/Runx2 and OPG/RANKL in tibial growth plate in TD broilers. **(A–D)** Principal component analysis (PCA) of the *BMP-2*, *Runx2*, *OPG* and *RANKL* gene expressions with an unsupervised pattern recognition method. **(E)** The mRNA expression of genes *BMP-2*, *Runx2*, *OPG*, *RANKL* and the ratio of *OPG/RANKL* at day 7, 14 and 21. **(F)** The heat map shows the mRNA levels of the bone-related genes. The results are represented as mean ± SD. a, b, c, and d represent significant differences between groups (*p* < 0.05).

PCA analysis was performed as an unsupervised pattern recognition method to explore the expression of bone-related genes in TD broilers and distinguish the potential effects of different doses of TFRD on bone-related genes (Figures 4A–D). PCA scores of *BMP-2*, *Runx2*, *OPG*, and *RANKL* genes showed that the distance between the TD and Con groups was greater than that between LTFRD and Con groups. Moreover, there was a dose-dependent effect in the TFRD-treated chicken groups. The TD + HTFRD group was farther away from the TD group, which suggests that supplementation of 500 mg/kg TFRD in drinking water has the best protective effect on protecting the bone injury of TD broilers.

The expression levels of *BMP-2/Runx2* and *OPG/RANKL* genes in TGP are displayed in Figure 4E. Compared with the Con group, the mRNA expression levels of *Runx2* on day 7 (*p* = 0.002) and day 21 (*p* = 0.020) were dramatically decreased in the TD group. Similarly, the expression levels of *BMP-2*, *OPG* and *OPG/RANKL* ratio were significantly decreased in the TD group during the experiment. Conversely, the mRNA expression of *RANKL* gene was significantly up-regulated throughout the experimental period in the TD group compared to the Con group (*p* < 0.001). Compared with the TD group, the three different doses of TFRD treatment clearly up-regulated the expression of *BMP-2* on day 14 (*p* = 0.710, *p* = 0.108, and *p* = 0.001, respectively) and day 21 (*p* = 0.357, *p* = 0.069, and *p* = 0.032, respectively), the expression of *Runx2* on day 14 (*p* = 0.466, *p* = 0.008, and *p* < 0.001, respectively) and day 21 (*p* = 0.017, *p* < 0.001, and *p* < 0.001, respectively), the expression of *OPG* on day 14 (*p* = 0.026, *p* = 0.010, and *p* = 0.002, respectively) and day 21 (*p* < 0.001, *p* < 0.001, and *p* < 0.001, respectively), and the ratio of *OPG/RANKL* on day 14 (*p* < 0.001, *p* < 0.001, and *p* = 0.001, respectively) and day 21 (*p* < 0.001, *p* < 0.001, and *p* < 0.001, respectively; Figure 4E). For the expression level of the *RANKL* gene, the three different doses of TFRD treatment could significantly reduce its expression on the TGP on day 14 and day 21 when compared with the TD group (*p* < 0.001, Figure 4E).

A heat map in Figure 4F further presented the expression levels of osteogenesis-related genes. The expression of bone resorption gene *RANKL* in TD broilers (red) was higher than that of Con group (blue), especially on days 14 and 21. Additionally, the expression of bone formation-related genes *OPG*, *BMP-2*, *Runx2* and *OPG/RANKL* ratio in TD broilers was evidently decreased. A high dose of TFRD had a better effect on restoring the expression of osteogenesis-related genes. Collectively, these results indicated that TFRD could ameliorate the expression levels of osteogenesis-related genes on the TGP of broiler chickens with TD.

### Effects of Total Flavonoids of *Rhizoma Drynariae* on the Protein Expression Levels of OPG/RANKL of Tibia Growth Plate in Broilers With Tibial Dyschondroplasia

**FIGURE 5:**
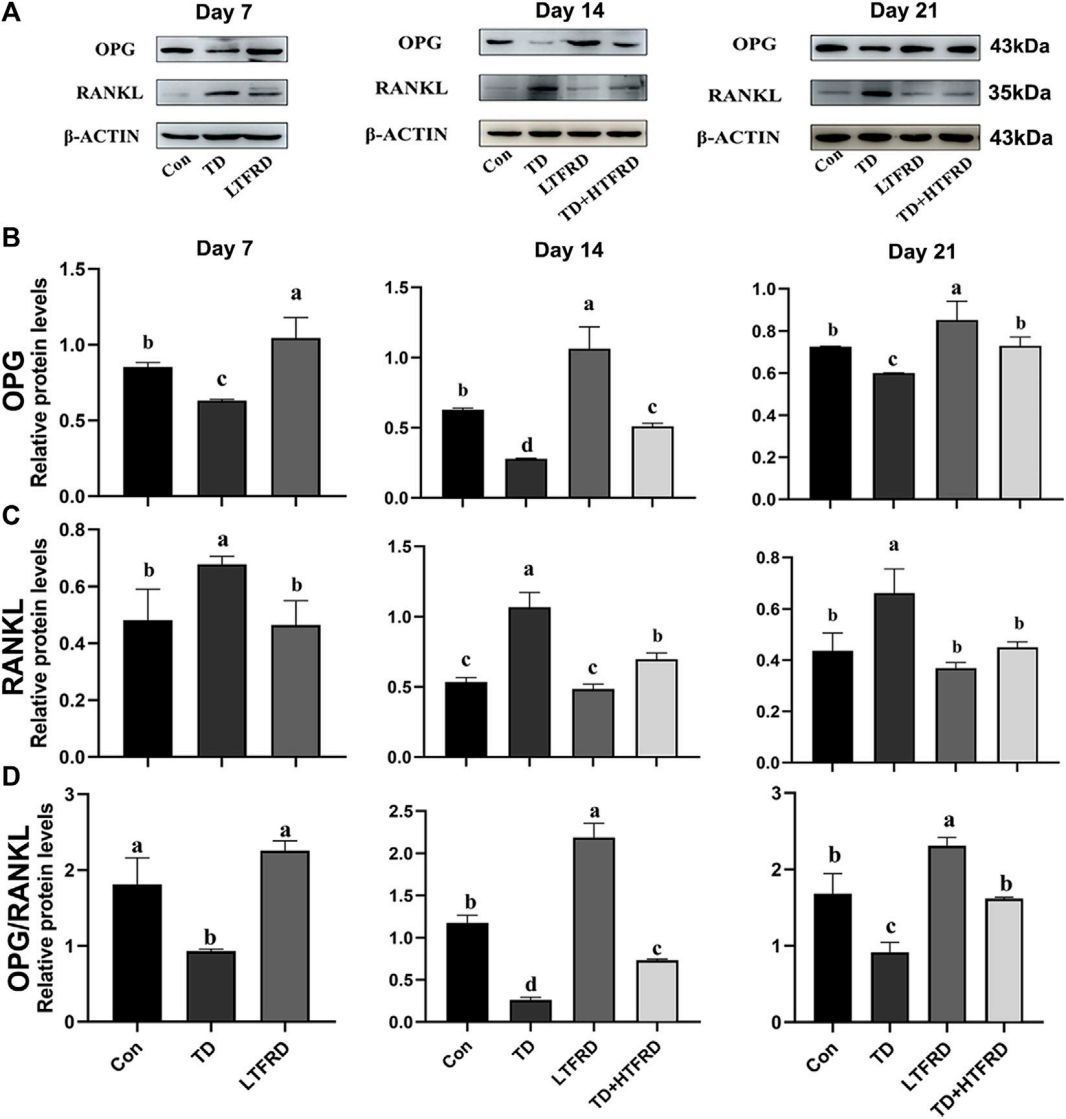
Effects of LTFRD on OPG/RANKL protein level of tibial growth plate in TD broilers. **(A)** The gray scale analysis of OPG, RANKL and β-ACTIN. **(B)** The protein level of OPG. **(C)** The protein level of RANKL. **(D)** The protein level of OPG/RANKL The results are represented as mean ± SD. a, b, c, and d represent significant differences between groups (*p* < 0.05).

The OPG and RANKL protein levels were assessed by western blotting analysis in the tibia of broiler chickens (Figure 5). The results demonstrated that the expression levels of OPG protein and OPG/RANKL ratio were significantly upregulated in the LTFRD group as compared to the Con group on day 7 (*p* < 0.001 and *p* = 0.129, respectively), day 14 (*p* < 0.001 and *p* < 0.001, respectively), and day 21 (*p* = 0.013 and *p* = 0.016, respectively). Compared with the Con group, the OPG protein and OPG/RANKL ratio were significantly down-regulated in the TD group on day 7 (*p* < 0.001 and *p* = 0.26, respectively), day 14 (*p* < 0.001 and *p* = 0.001, respectively), and day 21 (*p* = 0.005 and *p* = 0.008, respectively; Figures 5A,B,D). Moreover, it is found that TD + HTFRD group apparently increased the expression levels of OPG protein and the ratio of OPG/RANKL on days 14 (*p* = 0.002, and *p* = 0.008, respectively) and 21 (*p* = 0.007, and *p* = 0.011, respectively) as compared to TD group (Figures 5A,B). Additionally, the RANKL protein level was significantly up-regulated in the TGP of TD chicken during the experiment as compared to the Con group (*p* < 0.001). A high dose of TFRD treatment decreased the protein level of RANKL significantly as compared to TD group on day 14 and day 21 (*p* < 0.001 and *p* < 0.001, respectively). In addition, there was no difference in the expression of RANKL protein between the LTFRD and Con groups on day 14 and day 21 (*p* = 0.124 and *p* = 0.761, respectively, Figure 5C). These findings indicated that TFRD could activate the OPG/RANKL signaling pathway in broiler chickens with TD.

### Correlation Analysis Between Tibia Growth Parameters and Bone Formation-Related Indices in Broilers

**FIGURE 6:**
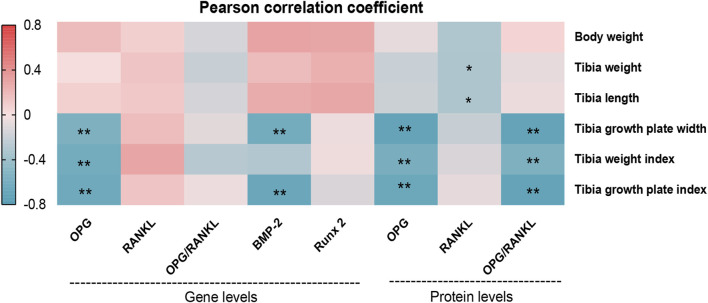
Correlation heat map analysis of tibial growth parameters and osteogenesis-related biological indicators in broilers. Note: The different rectangles are colored based on the Pearson correlation coefficients between tibia growth performance expression levels and bone formation-related indices. The intensity of color represents the degree of correlation, red represents positive correlation, blue represents negative correlation. *indicates significant correlation (*p* < 0.05), ***indicates highly significant correlation (*p* < 0.001).

To further analyze the potential relationship between osteogenesis-related biological indicators and tibial growth parameters, Pearson correlation analysis was performed (Figure 6). The results showed that body weight, tibia weight and tibia length were positively correlated with osteogenesis-related genes *OPG*, *BMP-2* and *Runx2*, but not significant; they were negatively correlated with RANKL protein (r = −0.291, −0.302, and −0.296; *p* = 0.052, 0.044, and 0.048, respectively). Interestingly, the TGP width and its index were negatively correlated with the gene expression of *BMP-2* (r = −0.582 and r = −0.634; *p* < 0.001 and *p* < 0.00, respectively) and *OPG* (r = −0.525 and r = −0.600; *p* < 0.001 and *p* < 0.001, respectively). Moreover, the protein expression of OPG and the protein expression of OPG/RANKL ratio were also negatively correlated with the TGP width (r = −0.669 and r = −0.627; *p* < 0.001 and *p* < 0.001, respectively) and its index (r = −0.669 and r = −0.675; *p* < 0.001 and *p* < 0.001, respectively). These results indicate that BMP-2/Runx2 and OPG/RANKL pathways may be involved in the regulation of tibia formation in TD broilers by affecting the function of tibia growth plate.

## Discussion

**FIGURE 7:**
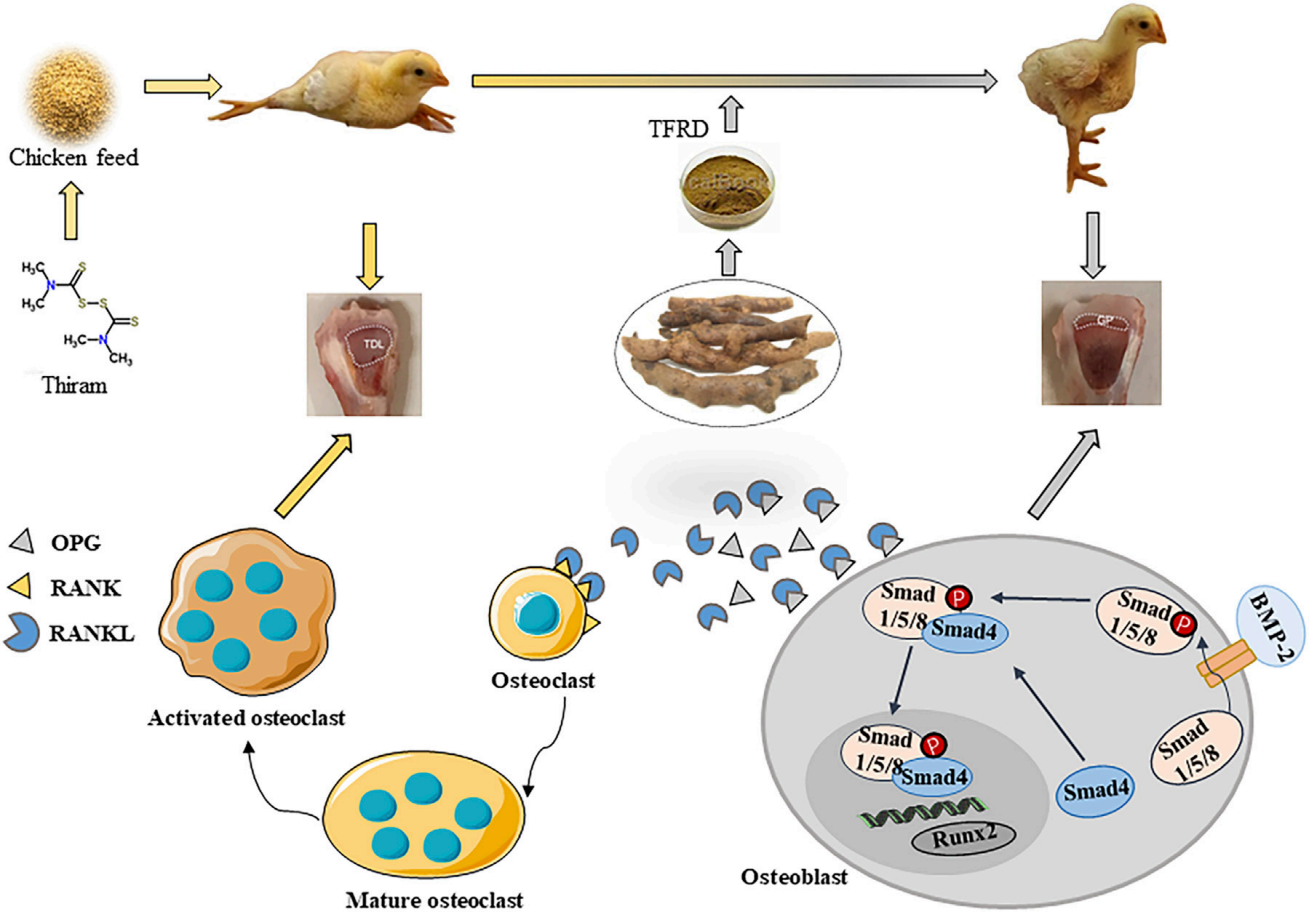
The schematic diagram of the protective mechanism of TFRD on TD broilers by regulating the OPG/RANKL expression.

Previous studies have indicated that TFRD, as a *Rhizoma Drynariae* extract*,* has been widely used in the prevention and treatment of many bone disorders including femoral head necrosis (Lv et al., 2021), osteoporosis (Wang et al., 2011), large tibial defects (Sun et al., 2021), by promoting osteogenesis, increasing bone mineral density, and preventing fracture in animal experiments (Shen et al., 2020). *In vitro* experiments have also shown that TFRD can increase the activity of osteoblasts, enhance the osteogenic effect of the membrane and improve the resistance of osteoblasts (Yu et al., 2021). In fast-growing poultry, tibial dyschondroplasia is an intractable tibiotarsal bone disorder that affects the proximal growth plate of the tibia bone (Huang et al., 2019a). In the present study, our results revealed that TFRD supplementation reduces lameness in TD broilers by increasing tibia growth parameters, reducing growth plate damage, improving growth plate vascular distribution, and regulating the expression of OPG/RANKL ratio, suggesting that TFRD had the effect on preventing and treating broiler chickens with TD (Figure 7).

Tibia growth performances have been widely used to evaluate the overall bone growth and development of broiler. The reduction of tibia weight and tibia indexes length indicates that the development of broiler bone is impeded. In the current study, the body weight gain of broilers in the TD group was significantly lower than those in the Con group. Additionally, the tibia weight and length were significantly decreased, while the tibia growth plate was markedly wider in TD broilers. TFRD as a TCM extract with bone strength (Zhang et al., 2017) confirm increased tibial volume fraction and accelerated fracture healing in fractured mice (Sun et al., 2021). In the present study, we demonstrated similar results that TFRD can increase body weight, tibia weight and tibia length, and decrease growth plate width, indicating TFRD plays a role in bone strengthening in TD broilers. The previous finding of Yao and coworkers (Yao et al., 2018) are consistent with the results of this study: TFRD also reduced mortality and accelerated recovery from lameness in TD broilers. These findings suggested that TFRD has a significant beneficial effect on the tibia growth performance of TD broilers.

The most direct reflection of the low tibia growth performance of the broiler is the transparent uncalcified embolism in the growth plate. In the present study, we investigated irregular transparent cartilage emboli with thickening in TD broilers (Figure 2A). These results are consistent with previous studies on thiram damage to growth plates of broilers (Zhang et al., 2018). The occurrence of transparent uncalcified embolism is mainly due to decreased angiogenesis in the tibial cartilage region, resulting in insufficient nutritional supply of osteogenic related cells, blocked metabolism and accumulation in the growth plate, resulting in calcification termination. TFRD has been shown to repair the properties of chondrocytes and promote the metabolism of osteogenic related cells, raise the proliferation of osteoblasts while inhibiting the activity of osteoclasts, thereby enhancing bone mass (Wei et al., 2017; Yao et al., 2018).

The TFRD was previously found to promote osteoblast activity, improve the accumulation of growth plate calcification and reduce growth plate width in TD broilers (Yao et al., 2018). In this experiment, the tibia growth plate of broilers in the LTFRD group showed good growth characteristics, and there was no difference in GP area between the LTFRD group and the normal group. Furthermore, TD lesion area of the growth plate of TD broilers treated with TFRD was significantly reduced, especially on day 21, and TD + HTFRD group had the best effect. These findings indicated that TFRD could be used as a long-term additive and has a good effect on improving osteoblast metabolism, reducing growth plate calcification in TD broilers.

To further study the occurrence and development of TD growth plate, we observed the vessels of the growth plate. There was almost no vascular infiltration in the growth plate in TD group, indicating that the growth plate of TD broilers was seriously damaged. Restoration of bone blood and nutrient supply is very important for tibial growth plate restoration of TD broilers. TCM can promote vascular regeneration and reconstruction (Huang et al., 2021b; Lv et al., 2021). TFRD, as a TCM widely used in bone diseases, has been proven that have the potential to promote vascular remodeling. Lv et al. (2021). treated avascular necrosis of the femoral head mice with TFRD and found that TFRD could improve the supply of femoral blood and nutrition, promote blood vessel regeneration, and strengthen the recovery of femoral osteonecrosis. Besides, TFRD stimulated vascular endothelial cell production and alleviated decreased vascular abundance in fractured mice (Shen et al., 2020). The previous results findings are similar to our study on TD broilers blood vessels, in the current study, vascular infiltration was enriched in Con and LTFRD groups, the vascular enrichment of TD broilers was gradually improved after TFRD treatment, and the effect was most obvious on day 21, which was the same as that of Con group. Therefore, TFRD can ameliorate the impaired growth plate development of TD broilers by promoting vascular growth.

The obstructed metabolism of osteoblasts and osteoclasts and accumulation in growth plate are the direct causes of transparent uncalcified embolism in TD broilers (Huang et al., 2019b). Promoting the maturation of osteoblasts and inhibiting differentiation of osteoclasts are important factors in alleviating TD broilers. BMP-2/Runx2 is a key signaling pathway that regulates osteoblast activity and plays a decisive role in bone formation. BMP-2 has strong osteoinductive potential *in vivo* and *in vitro*. It is an important stimulus for bone development (Soysa and Alles, 2016).

The Runx2 is a transcription factor important for the maturation of chondrocytes. It is also an important gene for osteoblast differentiation. TFRD has been proven to promote osteoblast activity and treat osteoporosis, bone fragility fractures, and bone loss (Lian and Stein., 2003; Wei et al., 2017). Previous studies have shown that TFRD can treat osteoporosis in rabbits and rats through BMP-2 related signaling pathway (Wong and Rabie, 2006; Wei et al., 2017). In TD broilers, TFRD improves the growth performance and restore normal vigor and significantly increases the expression of *BMP-2* and *Runx2* genes (Yao et al., 2018). We found that TFRD ameliorates TD-induced down-regulation of *BMP-2* and *Runx2* genes, suggesting that TFRD could enhance osteoblast activity and promote its expression during bone development in TD broilers. In addition, research has shown that TFRD can promote bone formation by activating the BMP-2/Smad signaling pathway and significantly improving the expression level of BMP-2/Runx2 in tibial vascular injury rats (Sun et al., 2021). These findings suggest that *BMP-2* and *Runx2* are important genes controlling tibial achondroplasia, and TFRD promotes osteoblast activity by regulating the expression of BMP-2 and Runx2, thereby ameliorating bone injury in TD broilers.

The OPG/RANKL is an important bone resorption regulator that inhibits osteoclast formation and activation. The OPG binds to the RANKL to form blocking competition for its receptor RANK, which is close to the differentiation and fusion of osteoclast precursors induced by osteoblasts. It regulates osteoclast formation, differentiation, maturation and apoptosis (Piemontese et al., 2016). Therefore, we detected the expression of OPG/RANKL relative content in TD broilers. The results exhibited that OPG expression was significantly down-regulated and RANKL expression was significantly upregulated in TD broilers compared with the Con group. This indicated that TD broiler osteoclast differentiation was strong, and tibia was seriously damaged. Several studies have shown that TFRD induces potential anti-osteoporosis activity by regulating targets in bone metabolic signaling pathways OPG/RANKL, and prevents and treats osteoporosis by inhibiting bone resorption or stimulating bone (Ibáñez, et al., 2019; Amin, et al., 2020; Ma, et al., 2020). Similar results were obtained in our study with TD broilers: TFRD treatment increased OPG expression level and decreased RANKL expression level of TD broilers, thereby improving tibial dysplasia in TD broilers. The high-dose group of TFRD had the best treatment effect on day 21. The ratio of OPG/RANKL can reflect the recovery rate of damaged bone. Our study found that TFRD supplementation significantly increased the OPG/RANKL ratio of TD broilers. Taken together, these results indicate that TFRD can inhibit the activity of osteoclasts by regulating the expression of OPG and RANKL, thus promoting bone development.

## Conclusion

The TFRD has a potential therapeutic effect on improving growth performance and promoting the vascular distribution of the tibial growth plate in TD broilers. Importantly, TFRD regulates bone formation via OPG/RANKL expression and thus diminishes lameness and recovers TD in broilers. Which provide a valuable reference for further exploring the effects of TFRD on poultry leg disease. And TFRD can be used as a potential target medicine to treat thiram-induced tibial dyschondroplasia.

## Data Availability

The raw data supporting the conclusion of this article will be made available by the authors, without undue reservation.

## Abbreviations

BMP-2, bone morphogenetic protein-2; GP, growth plate; OPG, osteoprotegerin; RANKL, receptor activator of nuclear factor kappa-B ligand; Runx2, runt-related transcription factor-2; TCM, traditional Chinese medicine; TD, tibial dyschondroplasia; TDL, tibial dyschondroplasias lesion; TFRD, total flavonoids of *Rhizoma Drynariae*; TGP, tibia growth plate.
