# Corrigendum: Total flavonoids of *Rhizoma Drynariae* ameliorate bone growth in experimentally induced tibial dyschondroplasia in chickens *via* regulation of OPG/RANKL axis

**DOI:** 10.3389/fphar.2022.969027

**Published:** 2022-08-11

**Authors:** Tingting Xu, Jingjing Zheng, WeiXing Jin, Lu Li, Luxi Lin, Aftab Shaukat, Chaodong Zhang, Qinqin Cao, Muhammad Ashraf, Shucheng Huang

**Affiliations:** ^1^ College of Veterinary Medicine, Henan Agricultural University, Zhengzhou, China; ^2^ Sanquan College of Xinxiang Medical University, Xinxiang, China; ^3^ National Center for International Research on Animal Genetics, Breeding and Reproduction (NCIRAGBR), Huazhong Agricultural University, Wuhan, China; ^4^ Livestock and Dairy Development Department, Pishin, Pakistan

**Keywords:** bone development, Chinese herbal medicine, leg disease, tibial dyschondroplasia, total flavonoids of *Rhizoma Drynariae*

In the published article, there was an error in Figure 5 as published. There is a misspelling of the group name in the Figure 5. The group names of protein grayscale images are shown as TFRD. The group of corrected protein grayscale images is named LTFRD. The corrected Figure 5 appears below.

**FIGURE 5:**
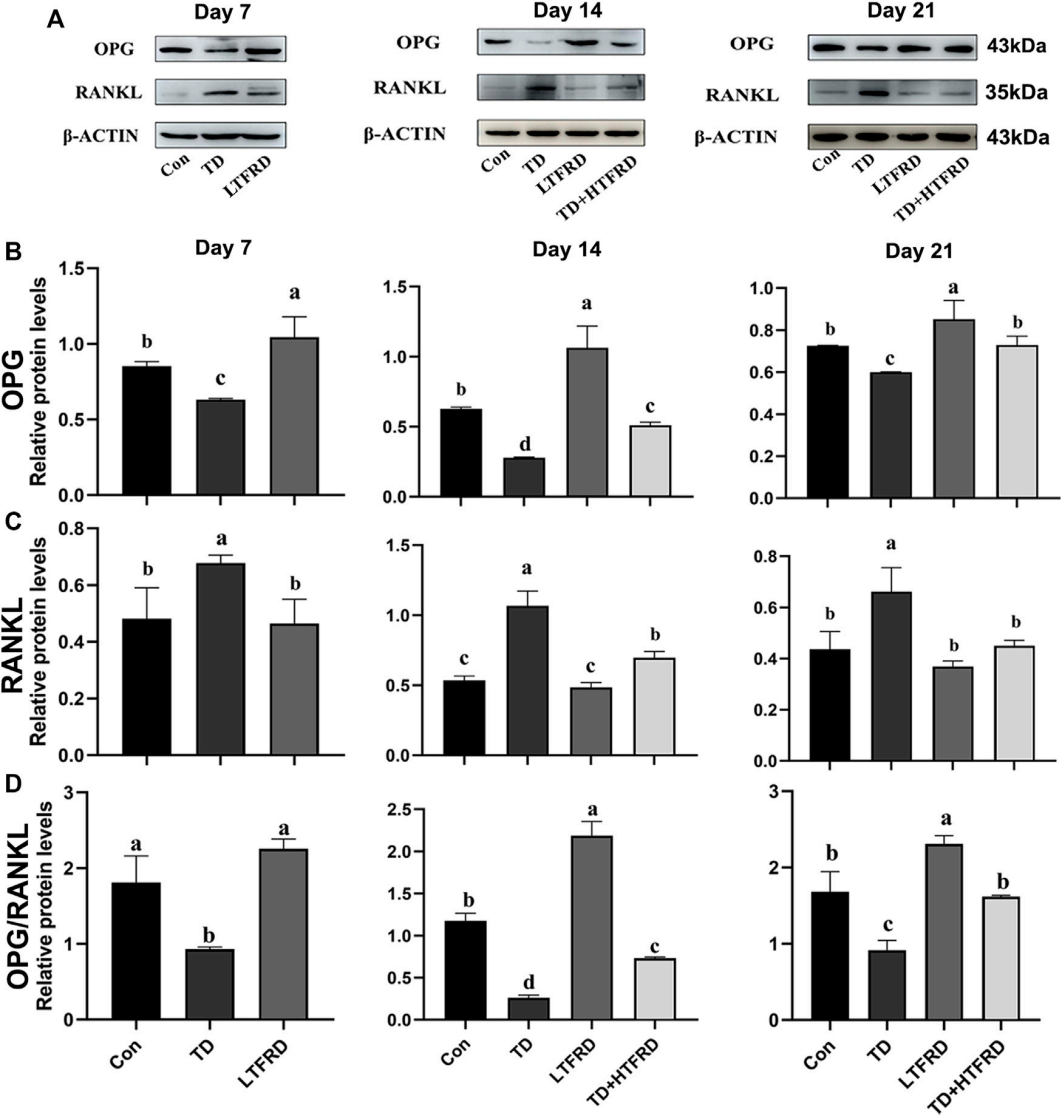
Effects of LTFRD on OPG/RANKL protein level of tibial growth plate in TD broilers. **(A)** The gray scale analysis of OPG, RANKL, and β-ACTIN. **(B)** The protein level of OPG. **(C)** The protein level of RANKL. **(D)** The protein level of OPG/RANKL The results are represented as mean ± SD. a, b, c, and d represent significant differences between groups (*p* < 0.05).

The authors apologize for this error and state that this does not change the scientific conclusions of the article in any way. The original article has been updated.

