# Supplementary material for: Total Flavonoids of Rhizoma Drynariae Ameliorate Bone Growth in Experimentally Induced Tibial Dyschondroplasia in Chickens via Regulation of OPG/RANKL Axis
Source: Front Pharmacol. 2022 May 26;13:881057. doi: 10.3389/fphar.2022.881057 (PMC9178197; doi:10.3389/fphar.2022.881057)
Supplement: Supplementary file 1 [file DataSheet1.docx]

Supplementary Material


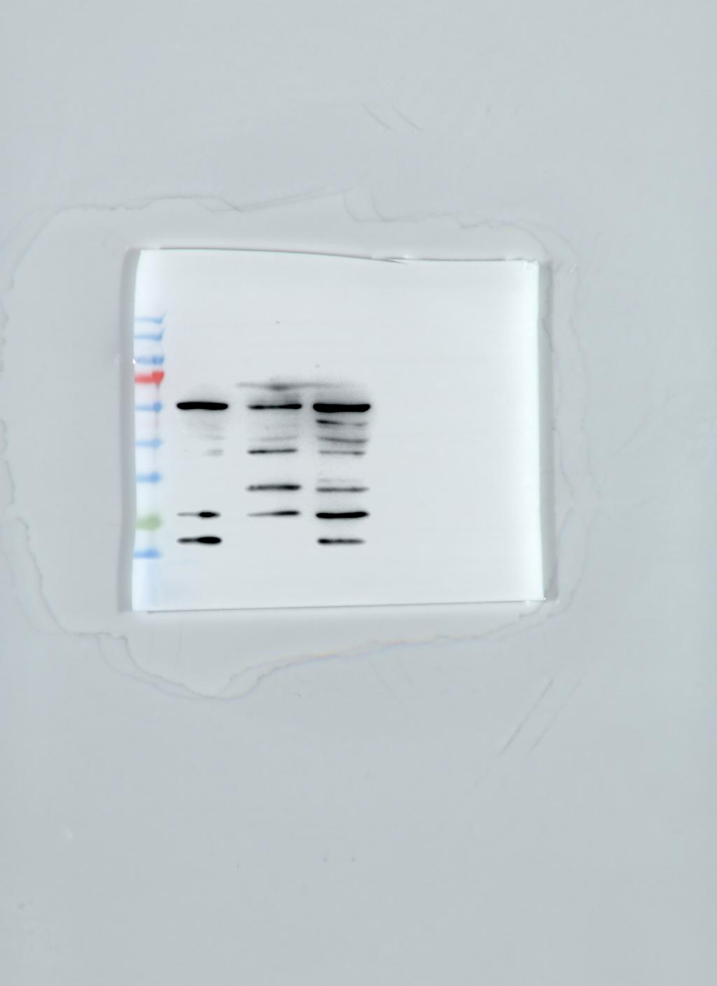

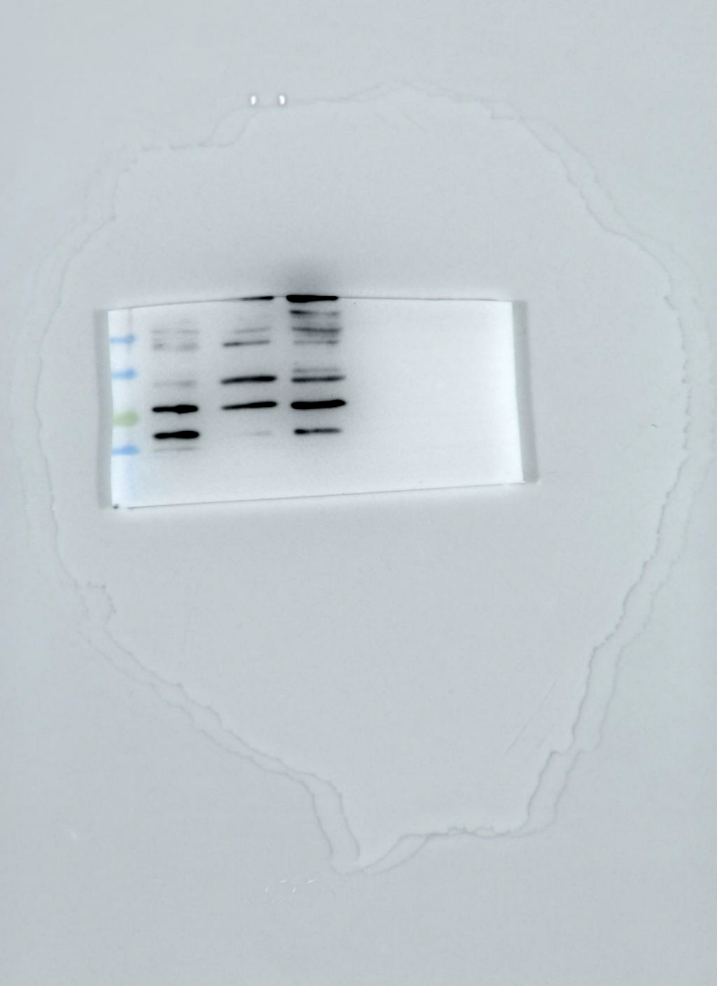

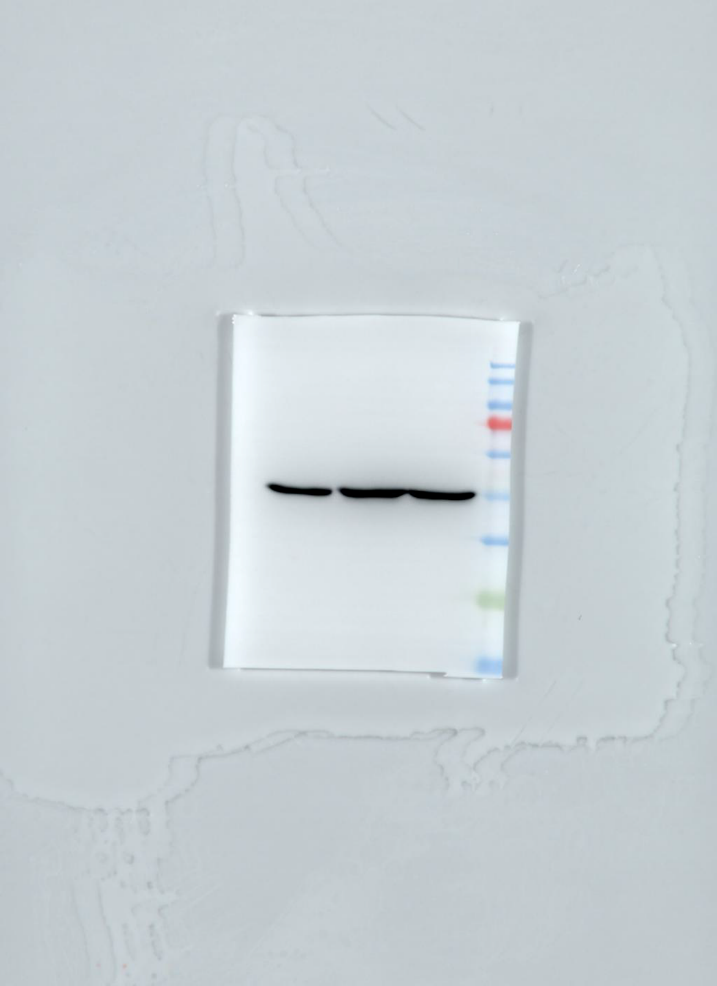


**Supplementary Figure 1.** The western blot images of OPG, RANKL and β-ACTIN on day 7. (Left to right)

**
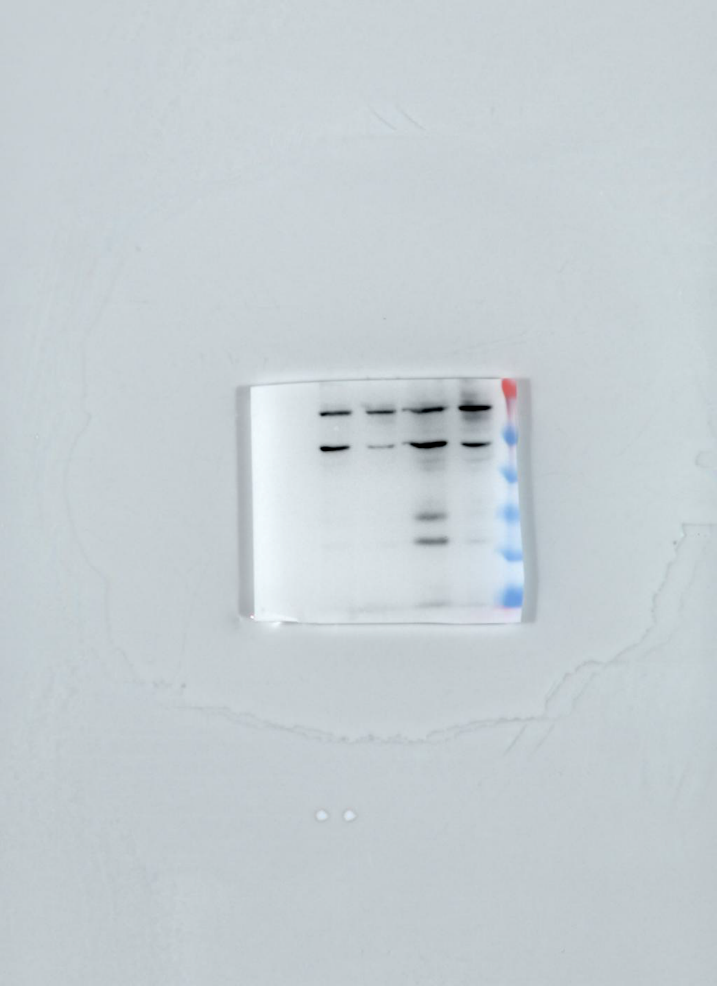

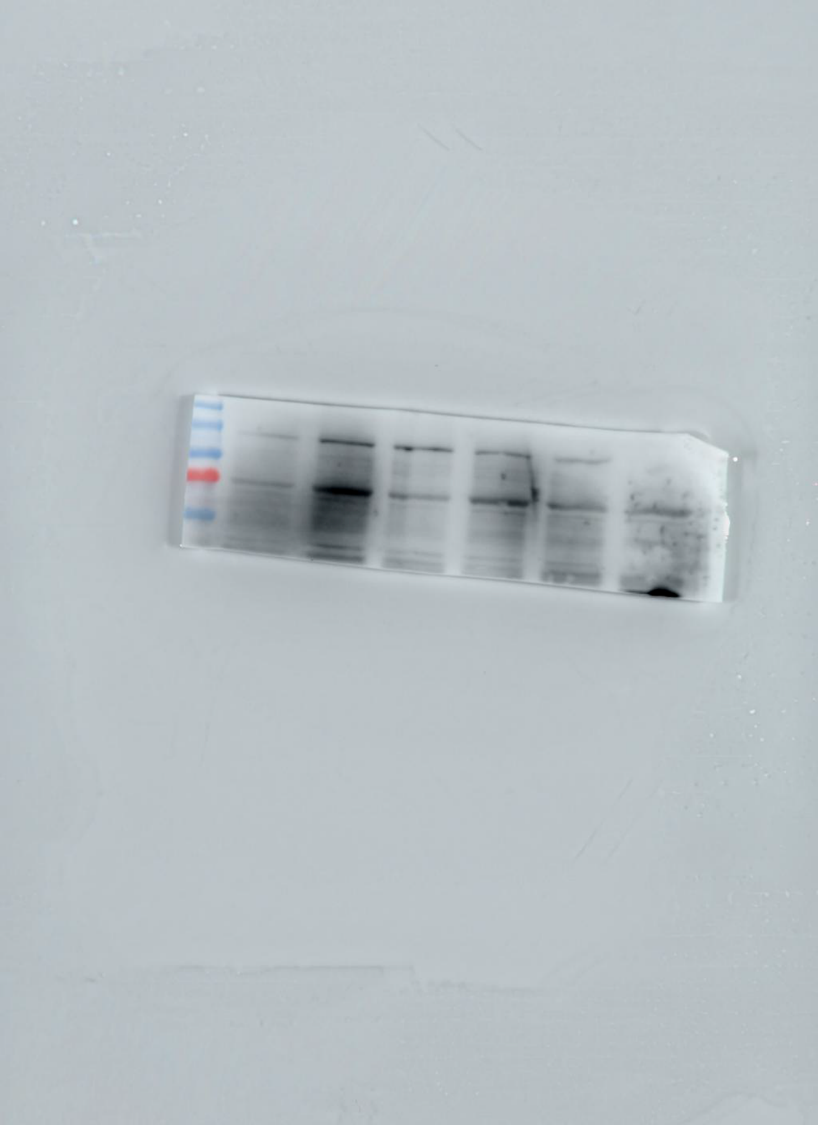

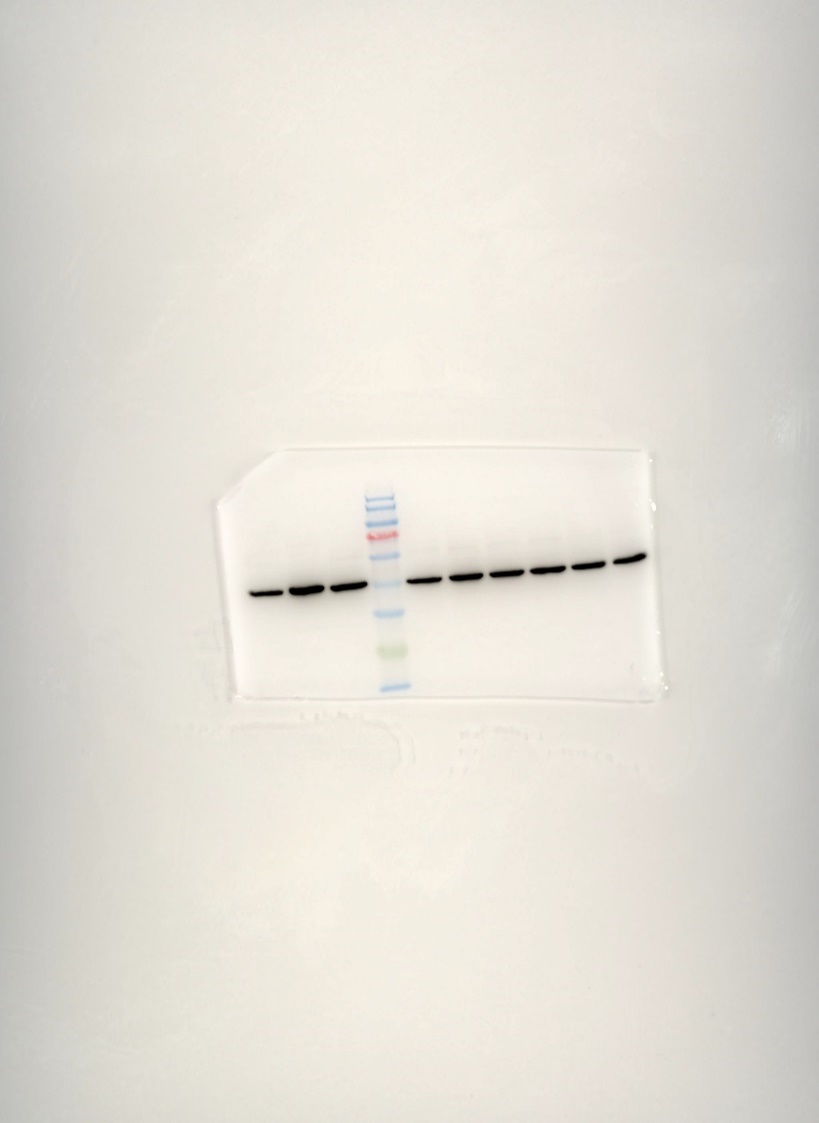
**

**Supplementary Figure 2.** The western blot images of OPG, RANKL and β-ACTIN on day 14. (Left to right)


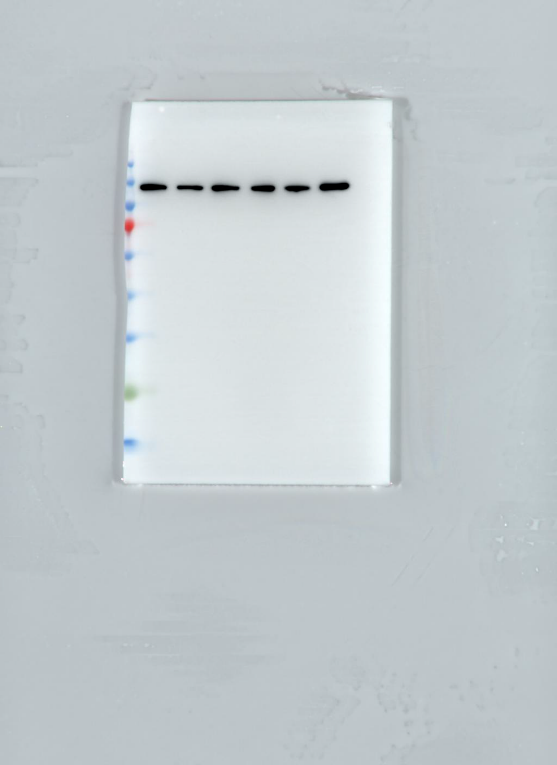

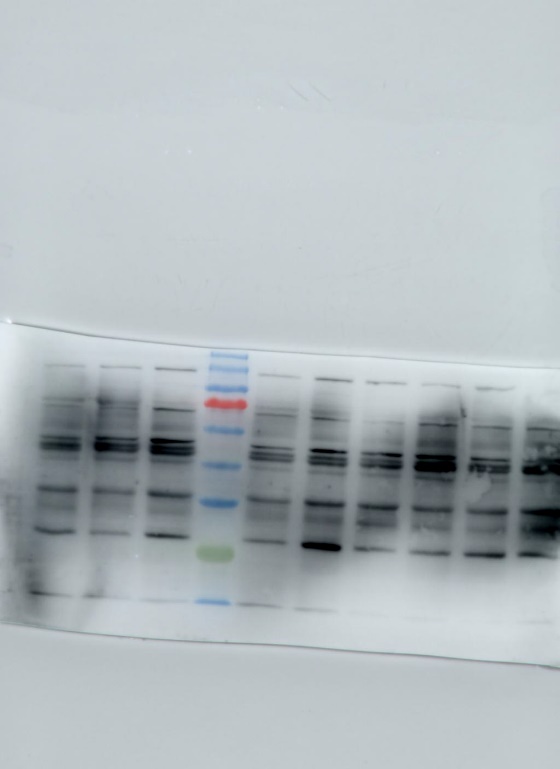

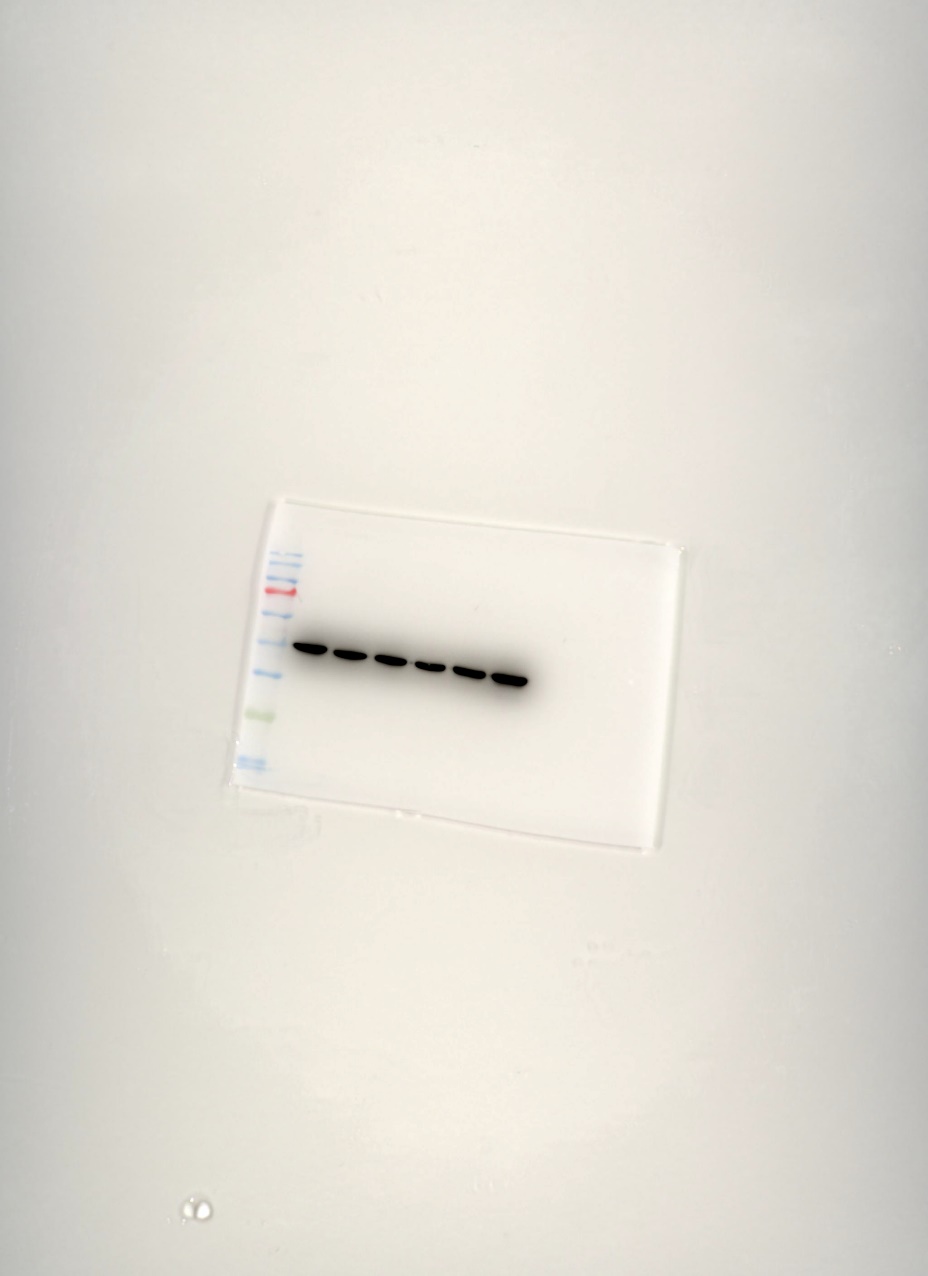


**Supplementary Figure 3.** The western blot images of OPG, RANKL and β-ACTIN on day 21. (Left to right)

**Supplementary Table 1:** The composition of TFRD

| **Chemical**  **composition** | **Content** | |
| --- | --- | --- |
|  | **μg/g** | **%** |
| Rutin | 401592 | 97.8233 |
| Quercetin | 7072.626 | 1.7228 |
| Quercetin 3-glucoside | 1119.576 | 0.2727 |
| Myricetin | 192.0882 | 0.0468 |
| Kaempferol | 149.7704 | 0.0365 |
| Astragalin | 148.6356 | 0.0362 |
| Genistin | 79.12365 | 0.0193 |
| Genistein | 47.70714 | 0.0116 |
| Taxifolin | 28.42508 | 0.0069 |
| Cynaroside | 22.18828 | 0.0054 |
| Formononetin | 18.52867 | 0.0045 |
| Luteolin | 18.25931 | 0.0044 |
| Isovitexin | 13.03135 | 0.0032 |
| Naringenin | 9.408299 | 0.0023 |
| Biochanin A | 6.306109 | 0.0015 |
| Vitexin | 4.016406 | 0.0010 |
| Daidzein | 2.543361 | 0.0006 |
| Apigenin | 1.512769 | 0.0004 |
| Liquiritigenin | 1.027148 | 0.0003 |
| Quercitrin | 1.00514 | 0.0002 |
| Glycitein | 0.086143 | 0.0000 |
| Chrysin | 0.014538 | 0.0000 |
